# Defining the elusive oncogenic role of the methyltransferase TMT1B

**DOI:** 10.3389/fonc.2023.1211540

**Published:** 2023-06-29

**Authors:** Sarah E. Denford, Brian T. Wilhelm

**Affiliations:** ^1^ Laboratory for High Throughput Biology, Institute for Research in Immunology and Cancer, Montréal, QC, Canada; ^2^ Department of Medicine, Faculty of Medicine, Université de Montréal, Montréal, QC, Canada

**Keywords:** TMT1B, METTL7B, thiol methyltransferase, protein methyltransferase, cancer biomarker

## Abstract

Methyltransferases are enzymes fundamental to a wide range of normal biological activities that can become dysregulated during oncogenesis. For instance, the recent description of the methyltransferase-like (METTL) family of enzymes, has demonstrated the importance of the N^6^-adenosine-methyltransferase (m^6^A) modification in transcripts in the context of malignant transformation. Because of their importance, numerous METTL family members have been biochemically characterized to identify their cellular substrates, however some members such as METTL7B, recently renamed TMT1B and which is the subject of this review, remain enigmatic. First identified in the stacked Golgi, TMT1B is also localized to the endoplasmic reticulum as well as lipid droplets and has been reported as being upregulated in a wide range of cancer types including lung cancer, gliomas, and leukemia. Interestingly, despite evidence that TMT1B might act on protein substrates, it has also been shown to act on small molecule alkyl thiol substrates such as hydrogen sulfide, and its loss has been found to affect cellular proliferation and migration. Here we review the current evidence for TMT1B’s activity, localization, and potential biological role in the context of both normal and cancerous cell types.

## Introduction: methyltransferases and the METTL family

Methyltransferases are a critical class of highly evolutionarily conserved enzymes capable of exerting a wide range of regulatory effects through their ability to transfer a methyl group onto a substrate. The human genome is estimated to contain 208 genes encoding methyltransferases (1) where the majority of these contain the canonical Rossmann-fold S-adenosyl-L-methionine (SAM) binding domain (2, 3). The activity of methyltransferases has been demonstrated against a broad range of substrates including nucleic acids (DNA/RNA), proteins, lipids, and small molecules (4–6). Arguably the most researched form of methylation is performed by DNA methyltransferases. Both the enzymes that methylate the DNA and the proteins that recognize this modification play critical roles in normal development through their ability to modulate transcriptional activity (e.g., gene silencing through the hypermethylation of gene promoter regions) (5). The methylation of protein substrates, notably histone tails, has also been investigated extensively and been shown to play an equally important role to DNA methylation in maintaining epigenetic regulation of genomes through a complex network of enzymatic “writers” and “readers”. Together, DNA and histone methylation provide a highly integrated system to functionalize genomes by controlling accessibility of the DNA for transcription that in turn influence cell fate and development through coordinated gene expression programs. More recently, another layer of methylation control over the transcriptome has been uncovered, namely RNA methylation. These base modifications, many of which are still being characterized, have been shown to have an equally broad range of impacts on mRNAs including their splicing, nuclear export, translation, and degradation (7). Given their diverse range of normal activities, it is perhaps not surprising that methyltransferase genes have been reported to have potential roles in many different cancers when dysregulated or mutated (4, 8, 9). In the context of malignancies, the identification of the substrate specificity of each enzyme is obviously of great interest, however current predictive methods are insufficiently accurate for this task for several reasons (3, 8). For instance, generating accurate protein structures where multiple subunits are involved in order to predict substrates remains challenging. In addition, the large degree of sequence variation in methyltransferase domains that target the same substrate means that there is a limited degree of certainty with respect to predicted substrates. As a result, the mechanistic activity of many methyltransferase enzymes in cancers remains unknown.

Within the SAM-dependent methyltransferases, one sub-group of enzymes whose importance in cancer has recently been elucidated are those encoded by the methyltransferase-like (METTL) genes. This family of enzymes contains 33 members which share little sequence conservation at the protein level outside of their SAM binding domain (8, 10, 11). The best-characterized members of the METTL family are METTL3 and METTL14, which form a heterodimer capable of catalyzing the N^6^-methyladenosine modification (m^6^A) on adenosine residues in RNA molecules (12). This modification can in turn be recognized by a wide range of modification “readers” that can influence transcript splicing, transport, stability, and translation (6). Other members of the METTL family have subsequently been shown to catalyze not only other nucleotide modifications such as N^4^-methylcytidine (METTL15) or O^2^-methyluracil (METTL19) but to also methylate protein substrates on different residues (METTL10, METTL11A/B, METTL20) (8, 11). The substrate and activity of other family members however, including Thiol Methyltransferase 1B (TMT1B, also known as **A**ssociated with **L**ipid **D**roplets **1**; ALDI, and **Met**hyl**t**ransferase-**L**ike **7B**; METTL7B), the focus of this review, remain poorly defined.

In the human genome, the TMT1B gene encodes a 244 amino acid protein that is most closely related to its putative paralog TMT1A, with the encoded proteins sharing 59% residue identity (13). However, despite the identical size of both proteins, and similar predicted localization to the endoplasmic reticulum (ER), the fact that TMT1B and TMT1A show greater similarity to their mouse orthologs (84% and 85% respectively) than to each other argue their functions have likely diverged over time. This argument is supported both by the biochemical studies discussed later and the divergent expression of both genes in normal tissue. While TMT1A is highly and ubiquitously expressed, TMT1B’s expression is largely restricted to the heart, liver, and colon (14). Given these differences, it is not possible to infer the function of TMT1B from its closest gene family relatives.

TMT1B was first identified in 2004 and characterized as a putative methyltransferase localized to the perinuclear face of the Golgi body in rat kidney cells (15). Later functional studies with purified proteins confirmed that TMT1B’s methyltransferase activity is SAM-dependent (13, 16, 17), while microscopy studies with cultured cells refined its subcellular localisation to the perinuclear face of the ER (13, 17, 18), and the surface of lipid droplets (LD) (18, 19), rather than the Golgi body (18, 20). Differential expression of TMT1B has been found to occur within several cancer types (4), including gliomas (21–25), non-small cell lung cancer (16, 23, 26–29), and acute myeloid leukemia (30, 31). TMT1B has been proposed as a biomarker for many of these cancers since its increased transcription and protein abundance is frequently correlated with an advanced TNM (Tumour, Node, Metastasis) stage of tumour development and poor patient overall survival (21–27, 29, 31–36), risk of recurrence (35) and drug resistance (16, 37). Despite these characterizations, the exact substrate of TMT1B, and its role in the various cancers where it is highly expressed, have not yet been defined. In this review we summarize the findings of several functional and biochemical studies of TMT1B to provide a comprehensive view of its activity and potential insight into its role in different cancers.

## The putative substrates of TMT1B

### TMT1B as a thiol methyltransferase

Perhaps the strongest evidence to date of TMT1B’s potential substrate was collected using *in vitro* methyltransferase assays with a purified His-GST tagged version of the enzyme (13). The results showed that while TMT1B had no activity against a range of commonly methylated metabolites including cysteine, glutathione, histamine, and 6-mercaptopurine, it could efficiently methylate other substrates such as dithiothreitol, 7α-thiospironolactone and hydrogen sulfide (H_2_S). The dependence of TMT1B on SAM for this methylation reaction was further validated through the introduction of a D98A mutation within its methyltransferase domain which effectively abolished its methyltransferase activity. Based on these results, the authors concluded that TMT1B acts as an alkyl thiol methyltransferase but not a thiopurine methyltransferase. While the biochemical evidence presented is clear, no measurements were performed *in vivo*, leaving open the possibility of other cellular ligands for TMT1B. Nevertheless, while the authors note physiological levels of hydrogen sulfide are disputed, their K_m_ measured for H_2_S is compatible with estimated cellular ranges (38). In addition, hydrogen sulfide’s connection to mitochondrial biogenesis and activity (39–44) could connect the function of TMT1B to cellular phenotypes when it is lost, however a precise role for H_2_S remain ambiguous. In mammalian cells, H_2_S can be produced by three enzymes including cystathionine γ-lyase, 3-mercaptopyruvate sulfur transferase, and cystathionine β-synthase and has been described as a gasotransmitter (45). Although toxic at high concentrations, at physiological concentrations, H_2_S can interact with DNA, protein and ROS metabolites thereby affecting a host of regulatory pathways involved in gene transcription and translation, as well as bioenergetics (44, 46). As noted, the effects of H_2_S on these pathways are largely dependent on its concentration, with excess H_2_S concentrations correlating with a decrease in ATP synthesis and an increase in cell death while cells with balanced H_2_S concentrations have increased proliferative and migratory abilities (44) as well as demonstrate antioxidant properties (42, 43). Finally, with respect to the methylation of H_2_S and the physiological importance of this molecule, this remains even more enigmatic with very little published work available to even guide speculation.

### TMT1B as a protein methyltransferase

Although TMT1B has only low levels of sequence conservation with other METTL family members that function as protein methyltransferases such as METTL10 (47) and METTL21B (48), evidence linking protein substrates to TMT1B has been reported. Indeed, when TMT1B was first identified as a substituent of the stacked Golgi fraction in rat liver cells, mass-spec analysis after incubation of this Golgi fraction with SAM detected di-methylation of arginine residues, although this activity could not be directly linked to TMT1B (15). In more recent studies examining neuronal development across multiple species using scRNA-seq, TMT1B was identified as a primate specific transcript expressed in a subset of neurons and subsequently performed unbiased proteomic studies to identify protein interaction partners of TMT1B (17). Four of these validated TMT1B interacting proteins (RTN3, RTN4, LRP1, and APP) were then purified and incubated with purified TMT1B and SAM to test for methyltransferase activity. Interestingly, all four proteins showed significant increases in methylation, demonstrating that a wide range of TMT1B interacting proteins can function as substrates for this enzyme. It is also worth noting that the study also found TMT1B-mediated methylation was modulated in the presence of a high lipid concentration due to the re-localization of TMT1B from the ER to LDs (17). In a similar finding, purified TMT1B exhibited little activity without being solubilized in 20% glycerol with the addition of dimyristoyl-sn-glycero-3-PG liposomes, suggesting that the lipid microenvironment may modulate TMT1B activity regardless of its substrate (13).

### TMT1B as an RNA methyltransferase

Given the well-characterized activity of different RNA-methylating METTL family members that have low sequence conservation, the possibility that TMT1B could also use RNA as a substrate is not implausible. This possibility has been investigated as part of a study of tyrosine kinase inhibitor (TKI) resistance in lung adenocarcinoma cells (16), however the evidence is somewhat circumstantial.

In this study the expression of TMT1B was found to be significantly higher in TKI-resistant cells along with the levels of m^6^A modification in the CDS and 3’-UTR of the transcripts of three ROS scavenging genes (HMOX1, SOD1, GPX4). Furthermore, the global levels of m^6^A, including on the HMOX1, SOD1, and GPX4 transcripts, were significantly decreased when resistant cells were treated with 3-Deazadenosine, an S-adenosyl-homocysteine hydrolase inhibitor. The correlated loss of m^6^A and resistance to gefitinib treatment therefore supports a link between mRNA methylation and TKI sensitivity. In addition, directly downregulating TMT1B levels with siRNAs also demonstrated a correlation between the loss TMT1B expression and the loss of drug resistance, which was independent of the levels of NRF2, an upstream regulator of ROS metabolic genes that are linked to drug resistance. Despite the clear differences in m^6^A levels when TMT1B levels were modulated, the expression levels of the other METTL members were not assessed during treatments with 3-Deazadenosine or siRNAs against TMT1B. This leaves open the possibility that the changes seen are not the direct consequence of TMT1B, but rather other METTL family members, a hypothesis supported by the observations that other METTL protein can add m^6^A modifications to the same ROS transcripts [e.g. METTL3 and GPX4 (49)].

## Functional role of TMT1B in cancer

Given the *in vitro* biochemical evidence for different potential substrates for TMT1B, the question arises whether any of the functional studies of TMT1B might provide further insight into its substrate, and therefore biological role. While more detailed studies would be required to precisely define its role, TMT1B activity could potentially touch on multiple cellular functions recognized as “hallmarks of cancer” such as involvement in proliferation, migration and invasion, the tumour immune microenvironment (22, 24), as well as cellular metabolism (13, 16). Because of its overexpression in a wide range of cancers, most of the published functional data is in this context and is broadly summarized in **Table 1** and briefly discussed here.

**Table 1:** A summary of cancers in which TMT1B activity is investigated with an interest in the effects of TMT1B expression on proliferation and migration.

Tissue Type	TMT1B Expression	Reference	Proliferation	Migration
*Clear cell renal cell carcinoma*	High	Li W, et al. (2021) (32)	Decrease (KD)	Decrease (KD)
	High	Xiong Y, et al. (2021) (22)	Decrease (KD)	Decrease (KD)
	High	Jiang Z, et al. (2021) (23)	Decrease (KD)	Decrease (KD)
	High	Xu L, et al. (2022) (21)	Decrease (KD)	–
*Non-small cell lung cancer*	High	Liu D, et al. (2020) (26)	Decrease (KD)	–
	High	Ali J, et al. (2020) (27)	Decrease (KD)	Decrease (KD)
	High	Li R, et al. (2022) (29)	Decrease (KD); Increase (OE)	Decrease (KD); Increase (OE)
	High	Li N, et al. (2022) (28)	Decrease (miRNA); Increase (OE)	Decrease (miRNA); Increase (OE)
*Papillary thyroid cancer*	High	Ye D, et al. (2019) (33)	Decrease (siRNA); Increase (OE)	Decrease (siRNA); Increase (OE)
	High	Zhu J, et al. (2022) (50)	Decrease (miRNA); Increase (OE)	Decrease (miRNA); Increase (OE)
*Glioma*	High	Chen X, et al. (2021) (24)	–	–
*Esophageal adenocarcinoma*	High	Dong Z, et al. (2019) (35)	–	–
*Endometrial carcinoma*	High	Wang A, Guo H, & Long Z (2021) (36)	–	–
*Acute myeloid leukemia*	High	Barabé F, et al. (2017) (30)	–	–
*Breast cancer*	Low	McKinnon CM & Mellor H (2017) (20)	–	No effect (OE)

Results from TMT1B-loss experiments with shRNA knockdown (KD), miRNA silencing (miRNA) or siRNA silencing (siRNA) are compared with those overexpressing TMT1B (OE). No data provided (–).

### Cellular proliferation

Several studies investigating TMT1B’s effects have highlighted a role in the regulation of cell proliferation in cancer cells. Following the loss of TMT1B expression in thyroid cancer cells, a significant decrease in proliferation was found, similar to effects seen in non-small cell lung cancer, clear cell renal cancer, and glioma cells (4, 21–23, 26–29, 32, 33, 50). Conversely, overexpression of TMT1B in thyroid cancer and lung adenocarcinoma cells significantly promoted cell proliferation (29, 50). TMT1B’s effect on proliferation has been seen in mouse models as well, with decreases in size and weight of xenograft tumours in BALB/c nude mice following subcutaneous implementation of TMT1B-silenced cells (16, 21, 26, 28, 32). Despite the data supporting TMT1B’s importance for cell proliferation, the mechanism by which it affects this process remains unclear. Experimental evidence shows that when TMT1B expression is lost or inhibited, cells enter cell-cycle arrest, with significant decreases of cells in both the S and G2/M phases (21, 26, 28, 32). Gene expression analysis in these studies noted a downregulation of cyclins (CCND1, CCNB1, CCNB2) and upregulation of cyclin-dependent kinase inhibitors (CDKN2C, CDKN2D) (26, 32). Similarly, in TMT1B-ablated lung adenocarcinoma cells, other proliferation-related genes such as PCLAF, CDC20, CDC25B, AURKB, MKI67, BIRC5 and HMGA1/2 were found to be downregulated (26). Finally, whether the loss of TMT1B leads to an increase in the rates of apoptosis remains unclear, with groups reporting both unchanged (26, 51) or increased apoptotic marker levels correlated with TMT1B expression.

### Migration and invasion

TMT1B activity in tumours is also linked to the cell’s ability to migrate and invade surrounding tissues. Following TMT1B ablation, trans-well cell migration assays in multiple solid cancer types demonstrated a decrease in motility and invasion (22, 23, 27–29, 32, 33). Wound healing assays in cells depleted of TMT1B showed a profound impairment in cell migration relative to control cells in both lung adenocarcinomas and gliomas (23, 27). Conversely, overexpression of TMT1B in thyroid cancer and lung adenocarcinoma significantly facilitated both migration and invasion (29, 33, 50).

TMT1B co-expression data shows enrichment in pathways involved in vasculogenesis (24, 25, 35) and cell migration (notably pathways related to the endothelial-mesenchymal transition; EMT) (23–26, 35). TMT1B ablation was found to inhibit the pro-migratory activity of TGF-β in thyroid cancer cells, where loss of TMT1B inhibited the decrease of E-cadherin and increase of N-cadherin typically seen during TGF-β-induced EMT (33). Additionally, protein and gene expression analysis of TMT1B-ablated cells highlighted an increase in E-cadherin (26, 32, 33) and decreases in Slug (32), Vimentin (23, 32), and N-cadherin (23, 32, 33) compared to wildtype cells. These observations therefore support the hypothesis that TMT1B plays a role in migration and invasion in cancer cells by modulating the expression of EMT-related genes. However, this hypothesis may depend on the level of TMT1B expression; while migration and invasion decrease following loss in cancers with high TMT1B expression, cancers with low TMT1B expression (e.g. breast cancer) actually show the opposite effect. In a series of knockdown and rescue experiments in HeLa and T47D cells, RhoBTB1, a Rho family GTPase, was found to regulate TMT1B expression (20). When either is depleted, cells developed Golgi fragmentation and the inability to polarize the Golgi to the leading edge of the cell, causing increased invasion, although no change in 2D migration was seen when TMT1B expression was increased (20).

## Localization of TMT1B

A further avenue through which to try and gain insight into the activity of TMT1B is through its sub-cellular localization. TMT1B was originally identified as a constituent of the Golgi complex and while later studies have linked its expression to the maintenance of Golgi integrity (20) this same study in HeLa cells found that TMT1B (and TMT1A) was localized to the ER, and not the Golgi. This discrepancy led the authors to speculate that the target of TMT1B might be a protein which shuttles between the Golgi and ER. It has been suggested that the largely hydrophobic N-terminus of TMT1B might play a role in directing its localization specifically to LDs. This view is supported by experiments with a GFP-tagged version of TMT1B expressed in COS cells where the protein was either in the membrane of the ER when cells were serum starved or in LDs when cells were pre-treated with fatty acids (FA) (18). As noted earlier, similar observations were made in neuronal cells (17), where TMT1B also translocated from the ER to LDs under high lipid conditions. LDs are thought to function as transport or communication hubs between organelles, including mitochondria (52, 53), through their transport of associated proteins (54). It is therefore possible to speculate that in the case of TMT1B, LDs might provide a mechanism for the enzyme to move to different subcellular locations. In this context, it is interesting that a mass-spectrometry based study looking at interacting proteins of METTL family members in HeLa cells only detected robust interaction between TMT1B and TMEM126A, an uncharacterized transmembrane mitochondrial protein (10). In the context of cancerous cells, where TMT1B often shows increased expression, lipid metabolism shows a definitive shift towards *de novo* FA synthesis—leading to a higher number of LDs (55)—as compared to exogenous accumulation of FAs in non-tumour cells in order to compensate for its higher energy consumption and increased cell division (56). Together, these observations suggest that the subcellular movement of TMT1B could potentially be linked to its possibly diverse substrates. At the same time, very few studies have conducted detailed studies of the subcellular movement of TMT1B, and none have been performed in the context of normal versus cancerous cells to elucidate whether its overexpression might lead to an aberrant localization and function specific to tumours.

## Discussion and conclusions

Despite the relatively sparse information on its biochemical substrates, functional studies characterizing the impact of the loss of expression of TMT1B have demonstrated its ability to impact multiple oncogenic pathways and play a role in cancer progression (8, 20). Overall, TMT1B expression in cancer is tied to pro-proliferation pathways, pro-migratory and pro-invasion functions, and finally to ROS scavenging and H_2_S metabolism. The question is therefore raised about whether TMT1B directly affects all these pathways (either alone or in a complex) or which of these effects may be indirect consequences. The answer to this question is likely linked to both its location in the cell as well as its interactions, whether with alkyl thiol molecules or proteins, in these subcellular locations.

**Figure 1:**
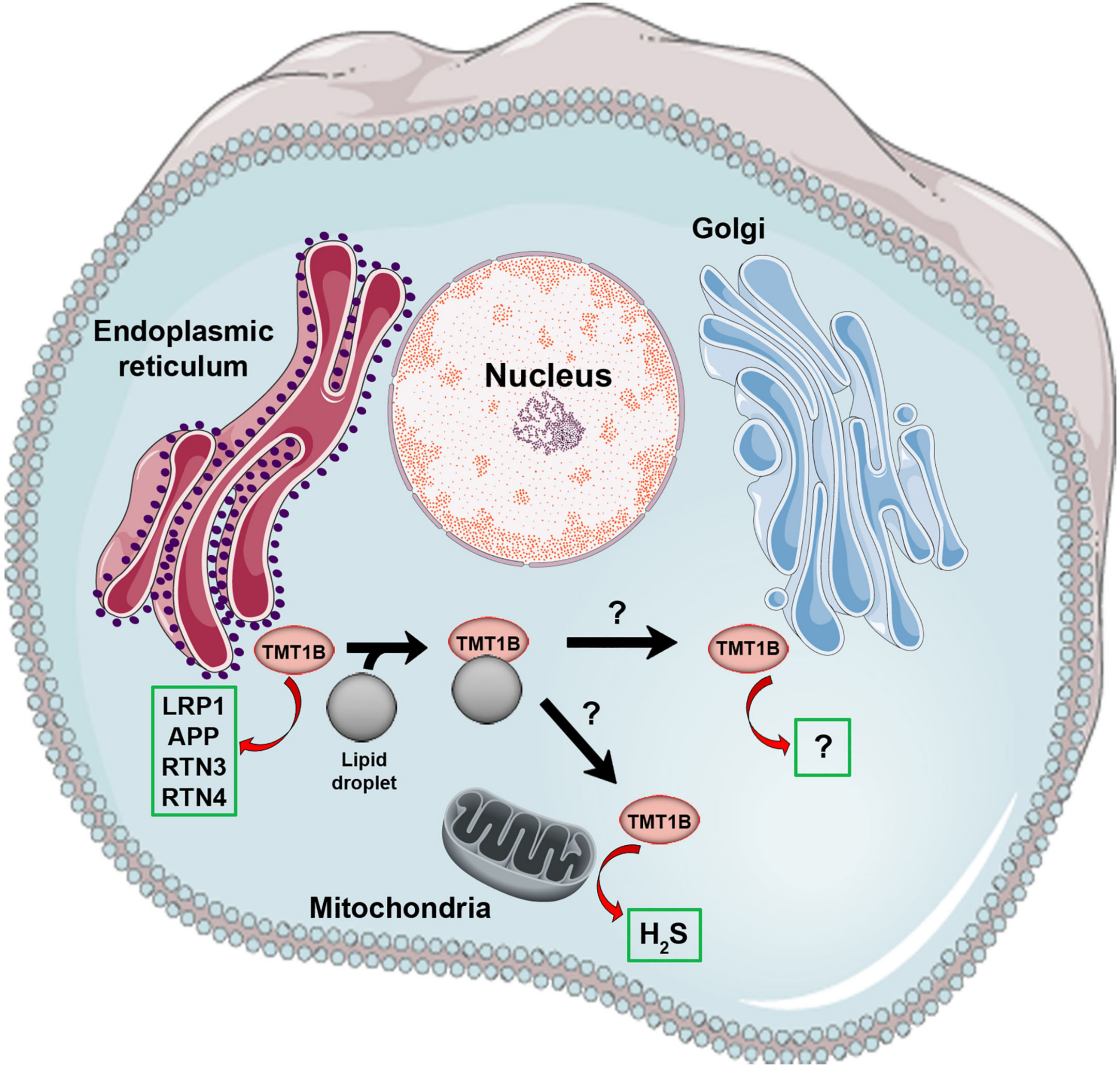
Known biochemical activities and sub-cellular localization of TMT1B. Shown is a cartoon summarizing current published data regarding the physical localization and enzymatic activities of TMT1B (shown as a pink circle). The sub-cellular movement of TMT1B, including its relocalization from endoplasmic reticulum to lipid droplets, is depicted with black arrows, however it is unknown whether lipid droplets facilitate the movement of TMT1B elsewhere in the cell (e.g. the Golgi apparatus where it was originally identified). Green boxes indicate the *in vitro* validated substrates of TMT1B published to date described in the main text. In the case of the Golgi apparatus, although methyltransferase activity was described in TMT1B fraction, the activity was not definitively connected to TMT1B.

There are at least two potential scenarios that could integrate the published data on TMT1B activity in cells and the phenotypes of its loss (see **Figure 1**). In the first case, its primary activity may be through the regulation of H_2_S metabolism, the direct methylation of which has been demonstrated. Through the maintenance of a balanced H_2_S environment, TMT1B could indirectly modulate ROS (42) and lipid (57) levels thus allowing for indirect regulation of cellular growth, migration and invasion (56, 58, 59). TMT1B’s role in H_2_S metabolism would provide an advantage to cancer cells when upregulated and could explain the protein interaction with mitochondrial membrane protein TMEM126A (10). The second possibility by which TMT1B can have such a range of impacts would be through its role as a protein methyltransferase. In this case, TMT1B could be shuttled between the Golgi complex, the ER and LDs (as previously shown) depending on the metabolic state of the cell, allowing for it to methylate a wide range of proteins depending on its current localization. These proteins could then, in turn, affect pathways involved in proliferation, migration and invasion, and ROS metabolism. It is important to note that these two scenarios are not mutually exclusive but rather TMT1B may methylate both thiol and protein substrates depending on circumstance. Given the divergent *in vitro* data published on its activity, additional *in vivo* targeted studies would be required to confirm published *in vitro* observations.

Regardless of its direct activity, it is clear that patients with TMT1B-high cancers generally show significant decreases in overall and disease-free survival (22–24). Additionally, regardless of if this is a direct cause or simply an effect of TMT1B expression, tyrosine kinase inhibitor resistance in lung adenocarcinoma patients was identified and the authors concluded that the resistance was due to TMT1B’s increased expression (16). The authors suggest that this might be due to TMT1B’s ties to an antioxidative pathway, although independent of its canonical regulator NRF2, since sensitivity to TKIs was reversed when cells were treated with ROS-scavenging inhibitors or TMT1B expression was ablated (16). This study suggests that from a therapeutic viewpoint of TMT1B over-expressing cancers, combinatory therapies would likely be required to block targets downstream of aberrant growth signals (e.g., tyrosine kinase receptor activation) such as ROS scavenging targets.

In conclusion, the identification of TMT1B as a biomarker in an increasing number of poor prognostic cancers recognizes this methyltransferase to be of importance in cancer progression. Continued comprehensive characterization of TMT1B’s activity, and the identification of its methylation targets will provide a better understanding of how this methyltransferase can impact such diverse and key pathways involved in cancers, and potentially provide mechanisms of inhibition for TMT1B-high cancers.

## Author contributions

SD: conception of study, literature search, data interpretation, writing of initial draft. BW: critical revision and editing of draft, preparation of figure, supervision of project. All authors contributed to the article and approved the submitted version.
